# Extracellular Matrix Enzymes and Immune Cell Biology

**DOI:** 10.3389/fmolb.2021.703868

**Published:** 2021-08-30

**Authors:** Meagan McMahon, Siying Ye, Jess Pedrina, Daniel Dlugolenski, John Stambas

**Affiliations:** Faculty of Health, School of Medicine, Deakin University, Geelong, VIC, Australia

**Keywords:** immunity, extracellular matrix, metalloproteinases, a disintegrin and metalloproteinases with thombospondin-1 motifs, a disintegrin and metalloproteinases

## Abstract

Remodelling of the extracellular matrix (ECM) by ECM metalloproteinases is increasingly being associated with regulation of immune cell function. ECM metalloproteinases, including Matrix Metalloproteinases (MMPs), A Disintegrin and Metalloproteinases (ADAMs) and ADAMs with Thombospondin-1 motifs (ADAMTS) play a vital role in pathogen defence and have been shown to influence migration of immune cells. This review provides a current summary of the role of ECM enzymes in immune cell migration and function and discusses opportunities and limitations for development of diagnostic and therapeutic strategies targeting metalloproteinase expression and activity in the context of infectious disease.

## Introduction

The extracellular matrix (ECM) forms the structural architecture surrounding cells and plays a key role in supporting tissue integrity and cellular functions. Remodelling of the ECM by metalloproteinase enzymes has being increasingly linked to immunity, especially immune cell migration (Bradley et al., 2012; McMahon et al., 2016; Chen et al., 2018; Boyd et al., 2020). Immune cell migration is critical for resolution of infectious disease. Large populations of mature immune cells do not normally reside at the site of disease and are instead located in the circulation or secondary lymphoid organs where they can be called upon when required. For example, following influenza virus infection, T cells become activated, proliferate in the mediastinal lymph node and then migrate to the lung to assist with clearance of virus. Using a murine model of influenza virus infection, one study has proposed that A Disintegrin-like and metalloproteinase with Thrombospondin-1 Motifs -5 (ADAMTS-5), an ECM metalloproteinase, facilitates migration of influenza virus-specific CD8^+^ T cells from the mediastinal lymph node (MLN) to the lung following infection (McMahon, Ye et al., 2016). In the absence of ADAMTS-5, viral clearance was disrupted. Conversely, a study using an alternative murine influenza virus infection model demonstrated that a closely related ECM enzyme, ADAMTS-4, when expressed in fibroblasts, contributed to increased lung damage following infection (Boyd, Allen et al., 2020). These studies emphasise the importance of the ECM and ECM remodelling in disease outcomes following virus infection and highlight the potential for development of ECM-based therapeutics. Recent advances in the fields of the ECM biology, metalloproteinase biology and immune cell migration, and the potential application of this new knowledge to the treatment of infectious disease will be discussed in detail throughout this review.

## The Extracellular Matrix Components

There are four major ECM components that contribute to structural integrity. These include proteoglycans, non-proteoglycan polysaccharides, fibres and multi-adhesive proteins. Each of these components has been shown to affect immune cell migration and will be discussed below.

### Proteoglycans

Proteoglycans are generated by most eukaryotic cells and consist of repeating units of covalently bound glycosaminoglycans (GAGs). GAGs provide adhesion points for a range of ECM molecules, including chemokines (Rot 1992; Wight 2002; Rot 2010). Chemokines have chemoattractant properties that drive leukocyte extravasation and migration through the ECM. The interaction between chemokines and GAGs is critical for immune cell recruitment and migration (Spillmann et al., 1998; Kuschert et al., 1999; Hirose, Kawashima et al., 2001; Li et al., 2002; Proudfoot et al., 2003). Versican, an ECM proteoglycan, binds C-C chemokine ligand (CCL)−2, −5, −8 and −21 (Hirose, Kawashima et al., 2001), all of which act as chemoattractants for immune cell migration (Murooka et al., 2008; Murphy 2010; Fang et al., 2012). GAGs also provide adhesion points for immune cells along migratory pathways (Gotte, 2003; Parish, 2006). Lumican, an ECM proteoglycan encoding a GAG domain, binds neutrophils helping them traverse the endothelial cell layer. It also promotes neutrophil migration via its interactions with β2-integrins (Lee et al., 2009).

ECM proteoglycans can also inhibit migration of immune cells. Versican has been shown to bind hyaluronan, a non-sulphated GAG widely distributed in connective tissue that acts to maintain ECM integrity (Suwan et al., 2009). Activation of T cells with poly I:C increased the viscosity of the ECM by facilitating versican-hyaluronan interactions, leading to the inhibition of CD4^+^ T cell migration (Evanko et al., 2012). In support of this proposition, analysis of versican expression in human cervical cancer samples has suggested that increased versican expression in stromal cells inhibited CD8^+^ T cell invasion, preventing CD8^+^ T cell clearance of tumorigenic cells (Gorter et al., 2010). Furthermore, a build-up of versican in the mediastinal lymph nodes of *Adamts5* ^−/−^ mice was associated with accumulation and poor migration of virus-specific CD8^+^ T cells to the periphery following influenza-virus infection (McMahon et al., 2016). In contrast, versican expression in a poly (I:C)-induced acute lung injury mouse model has been shown to encourage leucocyte infiltration and accumulation into lungs (Chang et al., 2014; Kang et al., 2016). These studies highlight the need for additional research to understand the contribution of ECM proteoglycans to immune cell migration.

### Non-Proteoglycan Polysaccharides

Hyaluronan is an ECM polysaccharide that consists of repeating units of glucuronic acid and N-acetyl glucosamine that can adhere to cell surface molecules such as CD44 (Lesley et al., 1994; Karvinen et al., 2003; Ruffell and Johnson, 2008; Suwan et al., 2009, Stephen P.; Evanko et al., 2012). The interaction between CD44 and hyaluronan facilitates mononuclear leukocyte adhesion to mucosal smooth muscle colon cells *in vitro* following inflammatory (poly I:C) stimulus (de la Motte et al., 2003). Hyaluronan also interacts with CD44 on the surface of T cells and is important for migration and activation of these cells (Lesley et al., 1994). However, the link between hyaluronan, cell surface molecules and immune cell migration needs to be further explored.

### Fibres

Collagen and elastin are the main structural fibres of the ECM, contributing to ECM stiffness and supporting tissue structure. Expression of collagen has been shown to alter migratory patterns of macrophages and T cells (Applegate et al., 1990; Li et al., 2003; Klose et al., 2013; Murray et al., 2013). Elastin can be degraded by metalloproteinase expressing macrophages and is critical for migration (Varga et al., 1997; Brassart et al., 1998; Hance et al., 2002; Antonicelli et al., 2007). Moreover, elastin degradation peptides encourage recruitment of mononuclear phagocytes (Varga et al., 1997; Brassart et al., 1998; Hance et al., 2002; Antonicelli et al., 2007). These studies highlight a key role for ECM fibres in immune cell migration. However, targeting ECM fibres for therapeutic use may prove difficult given the importance of these fibres in tissue structure.

### Multi-Adhesive Proteins

The ECM also contains ligands, such as fibronectin and laminin, that provide structural attachment sites for migrating immune cells. Laminin is a fibrous protein present within the basal lamina of epithelial tissue. It forms an intricate protein network for cellular contact to enhance structural integrity of tissues. Laminin α4 (a sub-type of laminin) knockout mice (*Lamα4* ^−/−^) have been successfully used to determine the contribution of this ECM component to immune cell migration. Croton-oil administration to the skin of *Lamα4* ^−/−^ mice showed reduced neutrophil and monocyte infiltration towards the inflammatory stimulus (Wondimu et al., 2004; Kenne et al., 2010). Moreover, in the absence of Laminin α4, reduced T cell infiltration into the brain was observed in an experimental autoimmune encephalomyelitis mouse model (Wu et al., 2009).

## Function, Structure and Regulation of Zinc-Dependent Metalloproteinases

### Function

In mammals, the zinc-dependent metalloproteinase (metzincin) superfamily includes 24 Matrix Metalloproteinases (MMPs) (Klein and Bischoff 2011), 40 A Disintegrin and Metalloproteinases (ADAMs) and 19 ADAMTS metalloproteinases (Brocker et al., 2009; Duffy et al., 2009; Mead and Apte, 2018). Of these, only 23 MMPs and 21 ADAMs have been identified in humans, while all 19 ADAMTS family members are found in humans (Raeeszadeh-Sarmazdeh et al., 2020). Metalloproteinases (MMPs, ADAMs and ADAMTSs) collectively cleave a large array of ECM substrates including proteoglycans, collagens and membrane-associated protein substrates such as cytokines (Black et al., 1997; Khatwa et al., 2010). Functionally, MMPs are responsible for regulating and degrading a variety of ECM components including collagen, elastin, and gelatin and contribute to regulation of cytokine expression (Ra and Parks, 2007; Siddhartha and Garg, 2021). Similarly, ADAMs cleave and release soluble factors like chemokines (Reiss and Saftig, 2009), mediate shedding of membrane-associated proteins into their active forms (i.e. TNF-α) (Black et al., 1997) or regulate gene expression through the generation of molecules that potentially act as transcription factors following intramembrane proteolysis and translocation to the nucleus (Reiss and Saftig, 2009). ADAMTSs are categorized based on cleavage substrates within the ECM–substrates include proteoglycans, pro-collagen N-pro-peptides, cartilage oligomeric matrix protein (COMP), and unknown or “orphan substrates” (Kelwick et al., 2015). A group of ADAMTS enzymes (ADAMTS−1, −4, −5, −8, −9, −15 and −20) can cleave aggrecan, versican, brevican, and neurocan and are termed “hyalectinases” (Abbaszade, Liu et al., 1999; Boerboom et al., 2011; Dancevic et al., 2013; Dancevic and McCulloch 2014). Cleavage of these ECM substrates allow metalloproteinases to play a key functional role in migration, proliferation and differentiation of cells. The role of metalloproteinases in the migration of immune cells will be discussed in detail below.

### Structure

**FIGURE 1:**
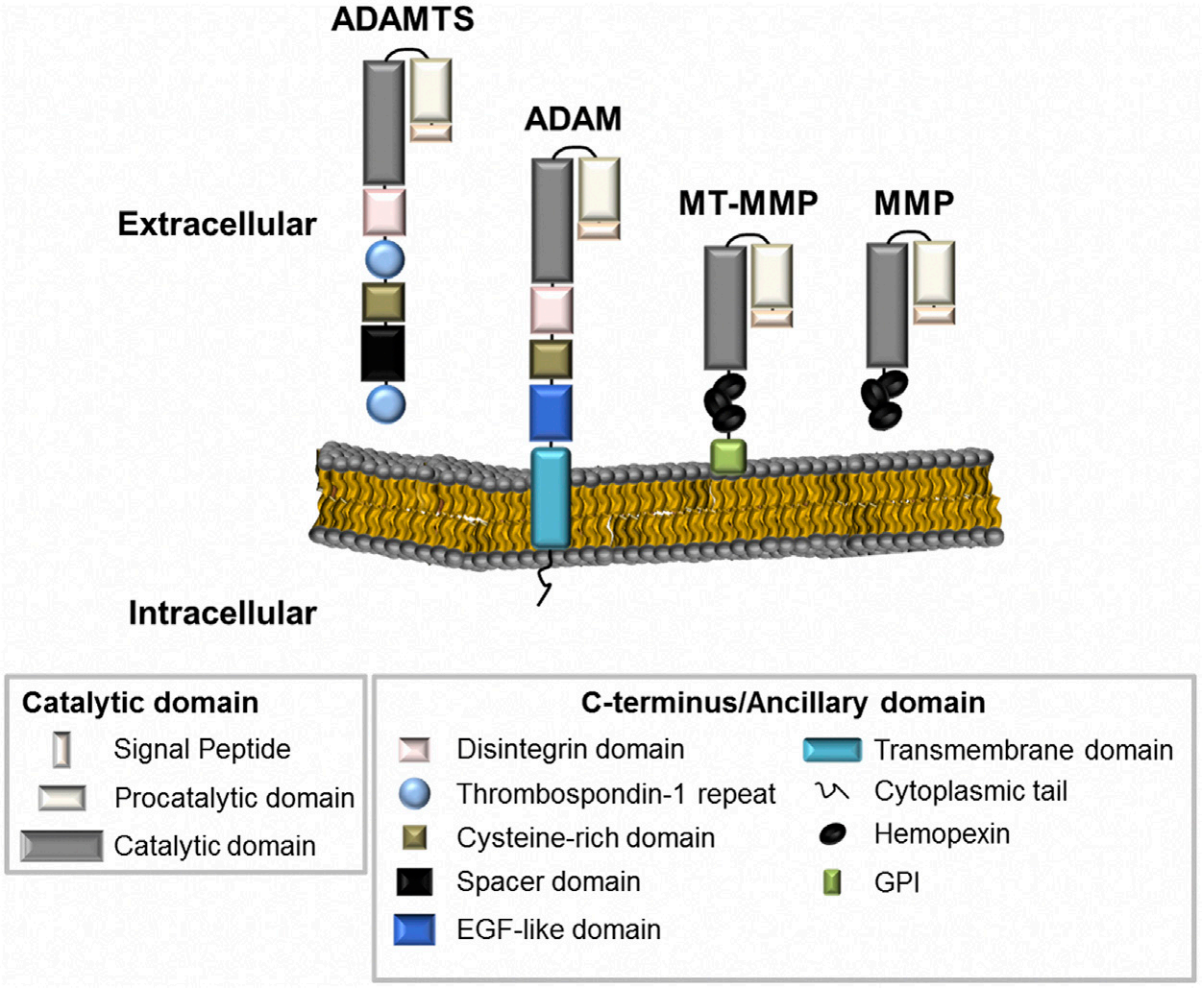
The metalloproteinase superfamily. The basic structural organisation of ADAMTS, ADAM and MMPs family members (including MMPs with a transmembrane domain, MT-MMP). Metalloproteinases generally contain similar proteinase domains. Differences in structure can be seen at the C-terminus or at the ancillary domain where ADAMTS enzymes contain Thrombospondin repeats; ADAMs are membrane-anchored through a transmembrane domain; and MMPs contain haemopexin-like regions.

All metzincins are synthesized as zymogens that contain a pro-domain and a catalytic domain for enzymatic activity, along with a distinctive C-terminus or ancillary domain (Massova et al., 1998; Nagase et al., 2006) (Figure 1). MMPs can be distinguished by the presence of a haemopexin-like domain that facilitates adhesion to their various substrates. MMPs are divided into three main categories–collagenases, gelatinases, and stromelysins based on their substrate specificity. They can also be categorized by the presence or absence of a transmembrane domain, allowing them to exist either as membrane-anchored or secreted metalloproteinases (Figure 1) (Klein and Bischoff, 2011). ADAMs and ADAMTS are distinguished from MMPs by the presence of a disintegrin or disintegrin-like domain, respectively. Metalloproteinase structure has been reviewed extensively and further information for these enzyme families can be found in the following review articles (Birkedal-Hansen, 1988; Birkedal-Hansen et al., 1993; Massova et al., 1998; Tang, 2001; Apte, 2009).

### Activation and Regulation

Metalloproteinases contain a conserved methionine residue at the active site and use a zinc ion for catalysis in enzymatic reactions (Bode et al., 1993). Activation of metalloproteinases varies depending on which type of zinc protease they represent; MMP, ADAM or ADAMTS. MMPs are synthesized and secreted as inactive enzymes. They remain in a latent state until they undergo catalytic activation by pro-protein convertases, such as furin (Ra and Parks, 2007). Catalytic activation of ADAMs occurs mostly intracellularly prior to secretion where they are found in their active form (Lum et al., 1998; Roghani et al., 1999; Kang et al., 2002). ADAMTSs share similarities with both ADAMs and MMPs and can be secreted in their inactive “pro” form or can be activated intracellularly and then secreted (Longpre et al., 2009; Kelwick et al., 2015). Metalloproteinase-mediated catalytic activity can be up-regulated or inhibited. Regulation and inhibition by host factors including reversion-inducing cysteine-rich protein with Kazal motifs, α-macroglobulin and the tissue inhibitors of metalloproteinases (TIMPs) has been characterized (Brew and Nagase, 2010). All four of the TIMP (TIMP-1–4) family members broadly inhibit MMPs (Greene et al., 1996; Ikonomidis et al., 2005; Jacobsen et al., 2008; Brew and Nagase, 2010; Kveiborg et al., 2010), with TIMP-3 inhibiting all MMPs, ADAM-10, −12, −17, −28 and −33, as well as ADAMTS−1, −2, −4 and −5 (Amour et al., 2000; Kashiwagi et al., 2001; Wang et al., 2006). These regulators of metalloproteinase activity can therefore be used to study disease processes.

## Metalloproteinase Regulation of Immune Cell Migration

As discussed above, ECM molecules are capable of inhibiting and supporting migration of immune cells. Immune cells (or relevant surrounding cells) express distinct metalloproteinases that interact with components of the ECM such as collagen and proteoglycans, to inhibit or promote immune cell migration.

### Neutrophils

Neutrophils secrete a range of pro-inflammatory molecules and immune mediators (reactive oxygen species, defensins and TNF-α), which have potent antiviral and antibacterial activity against infected cells. However, excessive neutrophil infiltration following inflammatory stimulus can lead to tissue damage and exacerbation of disease. Neutrophil infiltration into the lungs of mice infected with a laboratory adapted influenza virus (A/Puerto Rico/8/1934 (H1N1)) correlates with increased expression of MMP-2 and MMP-9, leading to pathology associated with enhanced cellular infiltrates and destruction of lung architecture (Bradley et al., 2012). In support of this, influenza virus infection results in MMP-9 secretion by neutrophils to facilitate infiltration into alveoli of the lung, which can be associated with poor disease outcomes in these mice (Wang et al., 2010; Narasaraju et al., 2011; Bradley et al., 2012). However, Bradley et al (2012), also demonstrate that MMP-9 expression is necessary for normal, protective neutrophil infiltration associated with viral clearance (Bradley et al., 2012).

### Macrophages

To enter tissue, macrophages must first traverse the basement membrane (Kelley et al., 2014; Tsuji et al., 2018). Macrophage-induced MMP-2, MMP-9, and MMP-14 (MT1-MMP) enzymatic activity facilitates infiltration and degradation of collagen in the basement membrane for a number of diseases, including fibrosis, vasculitis, and dermatitis. (Ray et al., 2004; Nishida et al., 2007; Gong et al., 2008; Klose et al., 2013; Watanabe et al., 2018). Indeed, depletion of plasminogen, which normally activates MMP-9 into its catalytically active form, results in reduced macrophage infiltration and a decreased likelihood of aortic aneurysm in a murine model of abdominal aortic aneurysm (Gong et al., 2008). In addition, reduced renal fibrosis was observed in *Mmp-2^−/−^* mice following unilateral ureteral obstruction (Du et al., 2012). MMP-14 has also been shown to be involved in macrophage infiltration in a murine model of contact dermatitis, where in *Mmp-14* ^−/−^ mice showed reduced macrophage infiltration at the site of dermatitis (Klose et al., 2013). These studies highlight the importance for MMPs in macrophage migration. In addition, related enzymes including ADAMs (ADAMs−8, −9, −15 and −19) and ADAMTS (ADAMTS−1, −4, −5 and −8) are found to be highly expressed in macrophage-rich areas in atherosclerosis. It is currently unclear if expression of these enzymes enhances macrophage infiltrating potential (Wågsäter et al., 2008; Salter et al., 2011).

### Dendritic Cells

Efficient dendritic cell (DC) migration is critical for initiating adaptive immune cell responses. In the absence of DC signaling, adaptive immune cell activation is severely impaired. The role of metalloproteinases in DC migration is currently under-studied. *In vitro* migration assays indicate that DCs isolated from *Mmp9* ^−/−^ mice show reduced migration when compared to their WT counterparts (Yen et al., 2008). To further expand on the role of *MMP-9* in DC migration, DC trafficking during allergen-induced airway inflammation in *Mmp9^−/−^* mice was assessed (Vermaelen et al., 2003). In the absence of MMP-9, inflammatory migration of DCs into the airway lumen was restricted, preventing the development of allergic airway inflammation. These studies highlight the importance of further defining the role of metalloproteinase mediated migration of DCs in acute and chronic disease.

### T Cells

Effector T cells do not normally reside at sites of disease. They are activated by DCs in lymph nodes and migrate to sites where they are required to perform their function. Indeed, migrating T cells in the high endothelial venules of lymph nodes require MMP-2 and -9 for normal migration (Faveeuw et al., 2001). Peripheral blood mononuclear cells derived from multiple sclerosis patients have been used to determine differences in migration of CD4^+^ T cell subpopulations *in vitro* using a transwell migration assay. T helper 1 (Th1) CD4^+^ T cells isolated from the aforementioned multiple sclerosis patients secreted higher amounts of MMP−2 and −9 when compared to Th2 CD4^+^ T cells, which was reflected through increased mobility in the transwell system (Abraham et al., 2005). Furthermore, inhibition of the Wnt pathway (a regulator of MMP−2 and −9 expression) results in reduced MMP−2 and −9 expression, leading to collagen accumulation and inhibition of T cell extravasation (Wu et al., 2007). The absence of MMP−2 and −9 in mice disrupts cleavage of collagen type IV and T cell movement through the ECM (Wu et al., 2007). Other ECM enzymes have also been shown to affect T cell migration. In human myeloma biopsy samples, one study has shown that samples containing high numbers of CD8^+^ T cells also demostrated elevated versican proteolysis via ADAMTS enzymes, suggesting these enzymes are important for T cell clearance of tumours (Hope et al., 2016). Mice lacking ADAMTS-5 (*Adamts5* ^−/−^ mice) show reduced movement of virus-specific CD8^+^ T cells following influenza virus infection (McMahon et al., 2016). Mechanistic analyses suggested that versican accumulation in the draining mediastinal lymph node interrupted egress of CD8^+^ T cells from the mediastinal lymph node to the periphery (McMahon et al., 2016). This result was further supported *in vitro,* where Jurkat CD4^+^ T human cells treated with an anti-ADAMTS5 antibody also showed impaired migration through versican in a transwell migration assay (McMahon et al., 2016). While the absence of ADAMTS-5 resulted in poor virus clearance and increased disease severity that was attributed to T cell migration (McMahon et al., 2016), a recent study by (Boyd et al., 2020) has demonstrated that the absence of a closely related family member, ADAMTS-4 in fibroblasts, leads to reduced lung immunopathology and improved lung function following lethal influenza virus infection (Boyd et al., 2020). This highlights the potential of these enzymes to influence outcomes of infection and emphasizes the need for further studies.

### Metalloproteinase-Mediated Cleavage of Cytokines to Promote Immune Cell Migration

Chemokines are a group of signaling molecules that are secreted by cells to promote migration of immune cells to the site of inflammation or disease. Neutrophils are typically the first cell type to respond to infection and infiltration may be supported by metalloproteinase activity. Nasal biopsies taken from allergic rhinitis patients, show that upregulated ADAM-12 in airway epithelial cells results in cleavage and release of the neutrophil chemoattractants, CXCL-1 and -8, from the ECM, which assist in the recruitment of neutrophils into the nasal cavity (Estrella et al., 2009). The use of *Mmp* knockout mouse models has also identified roles for MMP−7 and −8 in creating neutrophil chemotactic gradients. In *Mmp7* ^−/−^ mice, lung injury induced by bleomycin treatment resulted in a reduced transepithelial gradient of the chemokine KC (CXCL1), leading to reduced neutrophil influx (Li et al., 2002). Additionally, using a mouse model of acute colon injury Swee et al showed that the reduced neutrophil influx observed in *Mmp7* ^−/−^ mice due to changes in chemotactic gradients protected them from succumbing to colon injury, but that repair of colon tissue was delayed in these mice (Swee et al., 2008). These studies highlight the importance of metalloproteinase-mediated cleavage of cytokines in disease and exemplify an important role for this cleavage in promoting and controlling immune cell migration.

### Emerging and Future Areas of Interest

Metalloproteinases are attractive therapeutic targets where modulation of immune responses is required. They are essential for immune cell infiltration, cytokine regulation and tissue repair (Davey et al., 2011) and have the potential to be used as targets through the use of currently approved therapeutics or can be targeted by compounds under development; both of which have been eloquently reviewed by Raeeszadeh-Sarmazdeh et al., 2020 and Santamaria and de Groot, 2019 (Santamaria and de Groot, 2019; Raeeszadeh-Sarmazdeh et al., 2020). Of these potential therapeutics, the most widely studied include, small molecule inhibitors, antibody-based inhibitors, and tissue inhibitors of metalloproteinases (TIMPs), which are the natural regulators of metalloproteinase activity.

Small molecule inhibitors can elicit their inhibitory effects by targeting specific sites of the enzyme itself (catalytic or otherwise) and have been shown to target MMPs, ADAMs and ADAMTSs (Dufour et al., 2011; Remacle et al., 2012; Raeeszadeh-Sarmazdeh et al., 2020; Santamaria, 2020; Santamaria et al., 2021). While many of these compounds have shown potential in preclinical and clinical settings, off-target effects, lack of selectivity and specificity, and toxicity are all issues that need to be addressed (Cathcart et al., 2015; Mushtaq et al., 2018). In comparison to small molecule inhibitors, antibody-based inhibition is typically associated with less toxicity and immunogenicity (Fischer and Riedl 2019; Raeeszadeh-Sarmazdeh et al., 2020). As with small molecule inhibitors, antibodies have demonstrated varying degrees of success in targeting metalloproteinases in preclinical and clinical trials. Studies currently underway are assessing their use in the context of human health. (Dancevic and McCulloch, 2014; Santamaria et al., 2015; Shiraishi et al., 2016; Balchen et al., 2018; Santamaria and de Groot 2019; Raeeszadeh-Sarmazdeh et al., 2020). Manipulation of TIMPs may offer an alternative therapeutic approach. Given these proteins are natural regulators of MMPs, ADAMs and ADAMTSs, manipulation of TIMPs is a logical extension of inquiry. However, previous studies have highlighted both positive and negative outcomes to disease progression as reviewed by Raeeszadeh-Sarmazdeh et al., 2020 (Raeeszadeh-Sarmazdeh et al., 2020). This highlights the need for detailed understanding of TIMP regulation of metalloproteinase activity to inform development of future therapeutic options.

Conversely, as described throughout this review, enhanced expression of metalloproteinases may prove beneficial in certain disease states when increased immune cell migration is desired. While there are no therapeutics currently available that facilitate overexpression of these enzymes in clinical trial, to the best of our knowledge, the vast array of emerging inhibitors suggests that the same effect may be achieved via inhibition of the negative regulators themselves ie TIMPs. It is important to note however, that over-active metalloproteinase activity in other disease contexts e.g arthritis, has been shown to cause inflammation, leading to tissue destruction and poor outcomes in the host as observed in both human and animal studies (Davey et al., 2011; Syed et al., 2021).

Finally, although manipulating individual metalloproteinases seems reasonable, we are yet to fully elucidate how metalloproteinases act synergistically, and need to consider compensatory metalloproteinase activity in such instances. Moreover, many studies examining the function of multiple metalloproteinases in immunity have only been employed *in vitro* models, which do not take into account multi-faceted defence mechanisms elicited by a whole organism towards disease. Similarly most, *in vivo* metalloproteinase knock-out animal models have only dissected the role of individual metalloproteinases in disease processes although use of *Adamts7* ^−/−^ *xAdamts12* ^−/−^ mice and *Adamts4* ^−/−^ *xAdamts5* ^−/−^ mice have been reported (Majumdar et al., 2007; Mead et al., 2018). Further *in vivo* studies are required to understand how metalloproteinases act individually and synergistically before therapeutic intervention becomes a realistic treatment in the clinic.

## Conclusion

Metalloproteinases play an important role in regulating remodeling of the ECM to facilitate immune cell activity. Understanding how metalloproteinase activity is regulated and how family members act synergistically to influence immunity is critically important in order to develop novel therapeutic strategies.
